# Bio-Composite Filaments Based on Poly(Lactic Acid) and Cocoa Bean Shell Waste for Fused Filament Fabrication (FFF): Production, Characterization and 3D Printing

**DOI:** 10.3390/ma17061260

**Published:** 2024-03-08

**Authors:** Daniela Fico, Daniela Rizzo, Valentina De Carolis, Carola Esposito Corcione

**Affiliations:** 1 Department of Engineering for Innovation, University of Salento, Edificio P, Campus Ecotekne, s.p. 6 Lecce-Monteroni, 73100 Lecce, Italy; daniela.fico@unisalento.it (D.F.); valentina.decarolis@unisalento.it (V.D.C.); carola.corcione@unisalento.it (C.E.C.); 2 Italian National Council of Research-Institute of Heritage Sciences (CNR-ISPC), Campus Ecotekne, 73100 Lecce, Italy; 3 Department of Cultural Heritage, University of Salento, Via D. Birago 64, 73100 Lecce, Italy

**Keywords:** waste, cocoa shell, 3D printing, fused filament fabrication, PLA, circular economy

## Abstract

In this study, novel biocomposite filaments incorporating cocoa bean shell waste (CBSW) and poly(lactic acid) (PLA) were formulated for application in Fused Filament Fabrication (FFF) technology. CBSW, obtained from discarded chocolate processing remnants, was blended with PLA at concentrations of 5 and 10 wt.% to address the challenge of waste material disposal while offering eco-friendly composite biofilaments for FFF, thereby promoting resource conservation and supporting circular economy initiatives. A comprehensive analysis encompassing structural, morphological, thermal, and mechanical assessments of both raw materials and resultant products (filaments and 3D printed bars) was conducted. The findings reveal the presence of filler aggregates only in high concentrations of CBSW. However, no significant morphological or thermal changes were observed at either CBSW concentration (5 wt.% and 10 wt.%) and satisfactory printability was achieved. In addition, tensile tests on the 3D printed objects showed improved stiffness and load resistance in these samples at the highest CBSW concentrations. In addition, to demonstrate their practical application, several 3D prototypes (chocolate-shaped objects) were printed for presentation in the company’s shop window as a chocolate alternative; while retaining the sensory properties of the original cocoa, the mechanical properties were improved compared to the base raw material. Future research will focus on evaluating indicators relevant to the preservation of the biocomposite’s sensory properties and longevity.

## 1. Introduction

Respect for the environment and sustainability are issues of great concern today due to the growing problem of the disposal of industrial production waste. The circular economy (CE), a sustainable system that aims to reduce the consumption of raw materials and the production of waste by reintegrating them into a closed production cycle, underpins this research effort [1,2]. Disposal poses a significant challenge in the food industry, exacerbated by factors such as population growth. In particular, the escalating global demand for chocolate, a popular foodstuff in confectionery around the world, has led to a dramatic increase in the production of cocoa bean shell waste (CBSW) [3]. CBSW (100% cocoa bean shell) is typically repurposed as organic fertilizer, animal feed, or fuel, albeit with adverse environmental consequences [4]. In very few studies, CBSW is used as a renewable resource and as an additive to polymer matrix such as poly(lactic acid), PLA, which is known for its versatility and suitability in 3D printing with FFF technology [5,6]. Among the various methods for recycling waste materials, additive manufacturing (AM), or 3D printing, which facilitates the production of complex prototypes from computer-aided design (CAD) models while minimizing material waste [7,8], may be one of the most promising techniques. Its versatility extends to various industries, including advanced medicine and food processing, aligning with the transition from a linear to a circular economy model [9,10,11]. Previous research has demonstrated AM’s potential to reduce waste production, enhance product value, and promote sustainability [12,13,14]. Studies by Sanyang et al. [15], Papadopoulou et al. [16], and Tran et al. [17] have explored the use of cocoa waste in composite films and biocomposites, showcasing its potential as a natural filler with ecological benefits. Additionally, Leonard and Berrio [18] investigated PLA-based filaments incorporating cocoa husk for biomedical applications, noting improvements in compressive strength without altering thermal properties [19,20]. Andres J. Garcia-Brand et al. [21] pioneered a method for utilizing CBSW as reinforcement to enhance the bioactivity of synthetic polymeric materials in biomedical research. TGA and DSC thermograms unveiled the feasibility of employing various processing techniques, such as compression molding, extrusion, and potentially 3D printing. However, they did not explore the utilization of any 3D printing technology for fabricating 3D models using composite materials. In this study, biofilaments comprising PLA and CBSW at varying concentrations were developed and used to produce 3D models by FFF, offering a novel approach to waste recycling and reuse through FFF printing. The findings highlight the feasibility of integrating waste materials into the production cycle while emphasizing the importance of sustainable practices in modern manufacturing [22,23,24,25]. Based on this scenario, the aim of this study was to develop and characterize biocomposite filaments made from poly(lactic acid) and 5–10 wt.% of cocoa bean shell waste for FFF printing technology, from a CE perspective. The developed green filaments were subjected to morphological–structural, thermal and mechanical analyses. For the first time, the authors tested the feasibility of the FFF printing process to produce new 3D objects from waste materials, with the aim of permitting their integration into the same material production cycle (company-to-company). The work highlights the importance of sustainable practices in modern manufacturing while using solvent-free, inexpensive and easy-to-use methods.

## 2. Materials and Methods

The technical specifications of the materials utilized for the creation of composite biofilaments, manufacturing methodologies, and analytical techniques for structural, morphological, thermal, and mechanical characterization are outlined as follows.

### 2.1. Materials

The Ingeo 4043D poly(lactic acid) (NatureWorks LLC, Blair, NE, USA) employed as the polymer matrix for fabricating the composite filaments was obtained in pellet form. It possesses a density of 1.24 g cm^−3^ and a melt flow index (MFI) of 6 g/10 min at a temperature of 210 °C. PLA was utilized both independently to produce a virgin filament (100PLA) and as a polymer matrix for creating two biofilaments incorporating CBSW as fillers at 5 wt.% and 10 wt.%, respectively. Initially, the PLA pellets, with a diameter of approximately 5 mm (manufacturer’s data sheet), were stored in an oven at 60 °C for 24 h before being reduced into particles using the Retsch ZM 100 Ultracentrifugal mill (Retsch GmbH, Haan, Germany) equipped with a sieve with a mesh diameter of 0.75 mm.

The cocoa bean shell waste (CBSW) utilized in the production of the new composite filaments originates from the manufacturing residues of the company “Maglio Arte Dolciaria S.R.L.” (Maglie, Lecce, Apulia, Italy). Supplied by the company in the form of coarse-grained and heterogeneous powder, it exhibits the characteristic brown color of cocoa and a robust chocolate-like odor. Prior to utilization, the CBSW was subjected to storage in an oven at 60 °C for 24 h and subsequently reduced into particles using the Retsch ZM 100 Ultracentrifugal mill (Retsch GmbH, Haan, Germany) equipped with a sieve with a mesh diameter of 0.25 mm.

### 2.2. Production of Biofilaments for FFF

**Figure 1:**
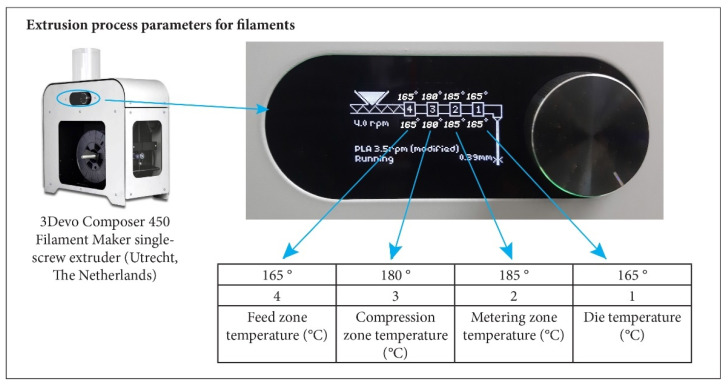
3Devo Composer 450 Filament Maker single-screw extruder with indication of different temperature zones.

The PLA and CBSW powders were initially combined manually at room temperature before being introduced into the extrusion chamber of the 3Devo Composer 450 Filament Maker single-screw extruder (Utrecht, The Netherlands,), as reported in Figure 1.

The parameters used to produce the filaments by the extrusion process are outlined in Table 1, while the labels and compositions of the materials used and developed are listed in Table 2.

### 2.3. 3D Printing

Three-dimensional samples were printed on the Creality CP-01 printer (Creality, London, UK) in accordance with the European standard used for the tensile tests ISO 527-2(2012) [26]. The following printing parameters were used: extrusion temperature 200 °C, plate temperature 50 °C, print speed 50 mm/s, fill 100% and fan speed 100%. The CAD model was developed using Fusion 360 software (Autodesk, San Rafael, CA, USA) and converted to a G-code file using Cura software, Ultimaker Cura version 5.1.1. (Ul-timaker B.V., Utrecht, The Netherlands). Finally, after studying the structural, thermal and mechanical properties of the biocomposites, some design objects (some traditional chocolate shapes) were printed for display in the window of the company that supplied the cocoa waste. The CAD models were developed using Fusion 360 software (Autodesk, San Rafael, CA, USA) and converted to Gcode files using Cura software, (Ultimaker B.V., Utrecht, The Netherlands). Prints were made with the Creality CP-01 printer (Creality, London, UK) with the following operating parameters: extrusion temperature 190 °C, plate temperature 50 °C, print speed 60 mm/s and infill 20%.

Three-dimensional objects (chocolate shaped objects) were also printed to demonstrate the printability of complex shapes with the recycled filaments. For this purpose, the BQ Hephestos 2 printer (BQ, Barcelona, España) was used with the following printing parameters: extrusion temperature 200 °C, plate temperature 0 °C, printing speed 60 mm/s and infill 20%.

A comprehensive list of all materials employed in this study is provided in Table 2.

### 2.4. Methods

The morphological characterization of the raw materials and the biofilaments produced was carried out using a scanning electron microscope (SEM), model Zeiss E Evo 40 (Oberkochen, Germany).

Fourier transform infrared spectroscopy (FTIR) analyses were performed on the raw materials (PLA and CBSW powders) to perform a preliminary chemical structural characterization. FTIR spectra were obtained on KBr pellets using a JASCO FT/IR 6300 spectrometer (Easton, MD) with a resolution of 4 cm^−1^, setting up 64 scans in the region between 4000 and 600 cm^−1^. Five spectra were considered for each replicate sample.

XRD measurements of the raw materials and biofilaments were performed with a Rigaku Ultima + diffractometer (Tokyo, Japan) using CuKα radiation (λ = 1.5418 Å) in the step scan mode, recorded in the 2θ range from 2–60°, with a step size of 0.02° and a step duration of 0.5 s. Five spectra were considered for each replicate sample.

DSC analysis (Mettler Toledo DSC1 StareSystem) was performed on raw materials, filaments and 3D printed rods to investigate thermal properties by measuring the glass transition temperature (T_g_), crystallization temperature (T_c_) and melting temperature (T_m_). The analyses were performed over a temperature range of 25 °C to 200 °C at a heating rate of 10 °C/min. 

According to the ISO 527-2(2012) standard [26], tensile tests were performed on the 3D printed sample using a Lloyd LR5K dynamometer (Lloyd Instruments Ltd., Bognor Regis, UK) at a strain rate of 1 mm/min. The grips used in these tests were wedge-type grips, the distance between grips was 58 mm, and the load cell was 5 kN. The dimensions of the specimen refer to the 1BA type, according to the standard. Five replicates were performed for each specimen.

Magnified images of the sections of the 3D printed tensile samples were obtained using the Dino Lite digital microscope instrument (AnMo Electronics Corporation, New Taipei City, Taiwan) to understand the morphology and adhesion of the different deposited layers.

## 3. Results and Discussion

### 3.1. Characterization of Raw Materials

The morphological–structural, chemical and thermal features of all raw materials used to produce biofilaments were investigated. The results of the SEM, FTIR, XRD and DSC analyses of the raw materials are shown below. 

**Figure 2:**
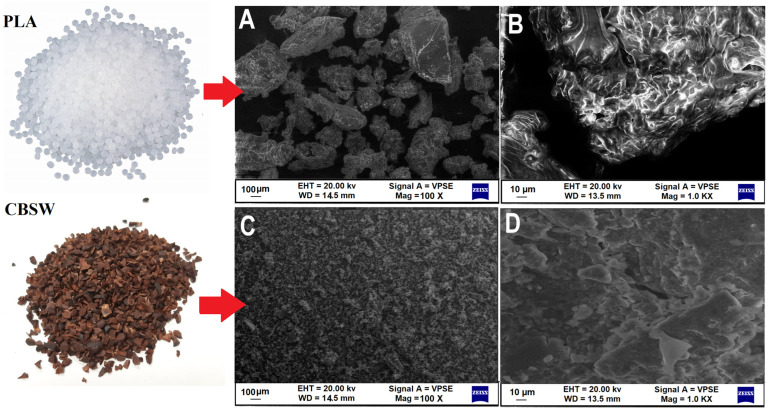
SEM images of raw materials: (**A**) PLA pellet magnification 100ϗ and (**B**) magnification 1Kϗ; (**C**) cocoa bean shell waste magnification 100ϗ and (**D**) magnification 1Kϗ.

After the milling process, SEM images (Figure 2A,B) at high magnifications (100ϗ and 1000ϗ) of the neat PLA particles show an irregular, square shape and a diameter up to about 500 μm. The cocoa bean shell waste (CBSW) was supplied in the form of a coarse-grained, inconstant powder, after grinding the cocoa bean shell. Furthermore, as visible from the SEM image (Figure 2C,D), the CBSW particle size varies from about 5 μm to about 200 μm.

**Figure 3:**
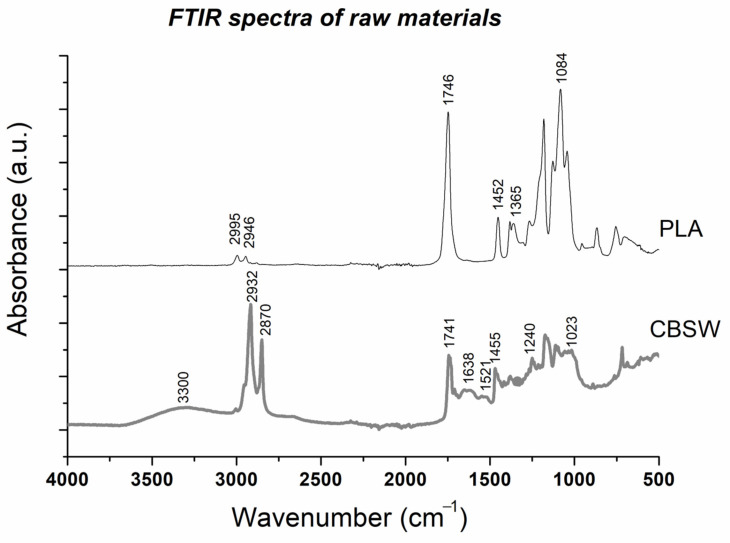
FTIR spectra of PLA pellet and cocoa bean shell waste and main infrared peaks.

The chemical characterization of the raw materials was carried out using FTIR spectroscopy. The infrared spectra of PLA powder and CBSW are shown in Figure 3.

The infrared spectrum of the PLA shows asymmetrical and symmetrical –CH_3_ and –CH_3_ stretching frequencies at 2995 cm^−1^ and 2946 cm^−1^, respectively, and a characteristic peak of C=O stretching at about 1746 cm^−1^ [27]. The peaks at 1452 cm^−1^ and 1365 cm^−1^ are associated with the asymmetric and symmetric bending vibrations of the –CH_3_ groups, respectively, while the band at about 1084 cm^−1^ corresponds to the C–O bond [27].

In the FTIR spectrum of the CBSW, the band present at around 3300 cm^−1^ is associated with OH groups, while the peaks at 2932 cm^−1^ and 2870 cm^−1^ correspond to the asymmetric and symmetric stretching vibrations of the C–H groups of cellulose and hemicellulose. The infrared peak at 1741 cm^−1^ corresponds to the C=O stretching of saturated esters, while the three infrared bands at approximately 1638 cm^−1^, 1521 cm^−1^ and 1401 cm^−1^ correspond to amide I, II and III, respectively. These bands can be associated with proteins, lignin and polysaccharides present in the biomass. Finally, the peaks at 1240 cm^−1^ and 1023 cm^−1^ are associated with holocellulose biomolecules [28].

**Figure 4:**
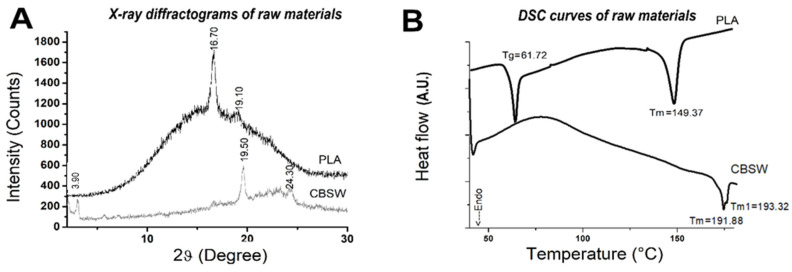
(**A**) XRD diffractograms and (**B**) DSC curve of raw materials.

In the XRD diffractogram of the PLA (Figure 4A), more defined diffraction peaks emerge at 2θ = 16.70° and at 2θ = 19.10° over a broad amorphous band, assigned to the crystalline plane (110) and (203), respectively [14]. The diffractogram of the CBSW sample (Figure 4A) shows peaks at 2θ = 3.09°, 19.5°, 21.20°, 22.02° (002), 23.11° and 24.30°, some of which are characteristic of crystalline polymorphs of cellulose [29,30]. The cocoa bean from which the powder was obtained is composed of about 60 wt.% water and holocellulose (cellulose, hemicellulose, pectin) and then lignin [29] and its XRD diffractogram is very similar to cocoa butter [31].

A DSC analysis was performed on raw materials to investigate thermal properties (Figure 4B). In the DSC curve of poly(lactic acid), the glass transition temperature (T_g_) was measured using the inflection point method with the STARe System version 11 software (Mettler Toledo, Milan, Italy) [12] and corresponds for PLA to 61.72 °C. In Figure 4B, it is also observed that the T_g_ is accompanied by an endothermic peak (relaxation enthalpy) due to the transformation of the polymer from the glassy to the liquid-viscous or rubbery state [32]. The DSC curve also shows a melting peak at temperature T_m_ of 149.37 and melting enthalpy ∆H_m_ of 19.90 J/g. In the present investigation, the DSC curves of CBSW samples were also examined. As with PLA, the average values obtained from the five measurements are shown in Figure 4B. The DSC analysis was carried out in the maximum temperature range commonly used in FFF printing with PLA filaments, i.e., between 25 and 200 °C. CBSW is a complex biomass consisting of several components, such as proteins, lignin and polysaccharides, as previously reported. Moreover, similar to woody biomass, the main polysaccharide components, consisting of hemicellulose, cellulose and lignin, manifest maximum endothermal peaks in different ranges according to the scientific literature [33,34]. Therefore, it can be concluded that the double endothermic peak present in the CBSW-DSC curve only refers to the thermal decomposition of hemicellulose and partially of cellulose.

### 3.2. Characterization of Biofilaments

**Figure 5:**
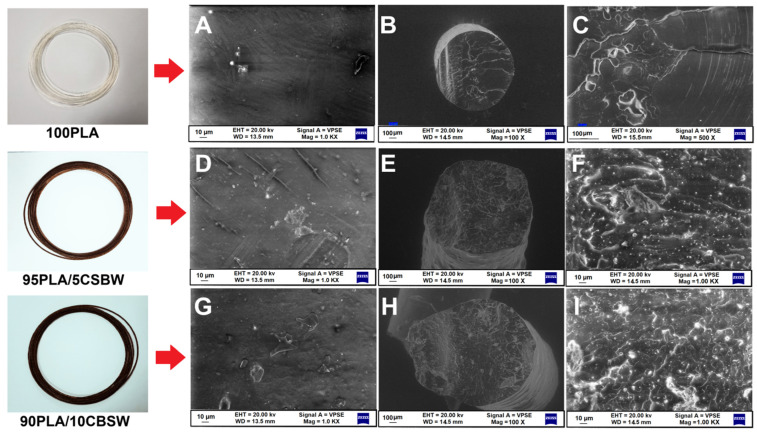
SEM images of filaments: (**A**) 100PLA external filament surface magnification 1.0Kϗ, (**B**) section magnification 100ϗ (**C**) section magnification 500ϗ; (**D**) 95PLA/5CSBW external filament surface magnification 1.0Kϗ, (**E**) section magnification 100ϗ, (**F**) section magnification 1.0Kϗ; (**G**) 90PLA/10CSBW external filament surface magnification 1.0Kϗ, (**H**) section magnification 100ϗ, (**I**) section magnification 1.0Kϗ.

SEM analyses were performed on the external surfaces of the composite filaments to investigate surface roughness and on the cross-section of filaments to verify homogeneity (Figure 5). The surface of the composite filament becomes slightly irregular after the addition of CBSW (Figure 5D–F), and some discontinuity areas are observed (Sample 95PLA/5CBSW). This phenomenon becomes more pronounced with the increase in the amount of filler added to the polymer matrix (Sample 90PLA/10CBSW), where the surface appears completely rough (Figure 5G–I). Furthermore, as shown by the cross-sectional images of the two produced FFF biofilaments, the cocoa particles aggregate in some places and these aggregates are even more visible in the filament containing 10 wt.% CBSW. The phenomenon appears to be attributable not only to a different diameter of the particles in the mixture (PLA approximately 500 µm measured by SEM and CBSW 5 µm to 1 mm diameter measured by SEM), but also to a different chemical nature that creates poor interfacial adhesion. This occurrence is already known in the literature for various PLA-based composites and waste fillers with different polarities and may indicate the possibility in the future of using plasticizer agents that improve the compatibility and adhesiveness of the different substances [35].

**Figure 6:**
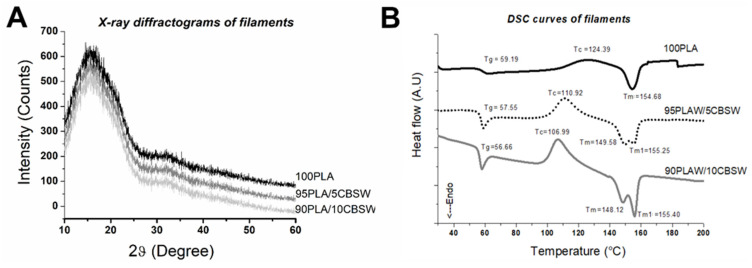
(**A**) XRD diffractograms and (**B**) DSC curves of filaments.

XRD spectra of filaments show only amorphous bands and a remarkable decrease in the intensity of diffraction peaks relative to the raw materials (Figure 6A). However, no major differences are shown between the 100PLA filament and the 95PLA/5CBSW and 90PLA/10CBSW filaments, indicating that the reticular parameters of the PLA crystal structure remained unchanged after coextrusion of the polymer with CBSW. Similar observations were also reported by Tran et al. 2017 for poly(ε-caprolactone) matrix biocomposites [17]. Therefore, to obtain information on the variation in the thermal properties of composite filaments compared to pure materials, a DSC analysis was performed (Figure 6B). The DSC thermograms of the PLA-based biofilaments or PLA and CBSW-based filaments are shown in Figure 5B. The glass temperatures (T_g_) of all materials were measured using the inflection point: the T_g_ coincides with the point at which the second derivative is equal to zero. The T_g_ measured for the 100PLA sample was 59.19 °C, a value similar to that reported in the literature [1] and slightly lower than that of the starting pellet.

Composite filaments show a lower T_g_ than the PLA filament. This suggests that the cocoa particles interacted with the PLA on a molecular scale. However, the main differences due to this interaction process between the polymer chains and the filler are found in the crystallization (T_c_) and melting (T_m_) temperatures. The crystallization temperature T_c_ in the 95PLA/5CBSW and 90PLA/10CBSW filaments compared to the 100PLA filament having a T_c_ of 124.39 °C decreases to 110.92 °C and 106.99 °C, respectively. Furthermore, the melting process seems to be particularly influenced by the presence of the CBSW filler, which leads to the development of a double melt peak (T_m_ and T_m1_) in composite biofilaments, which is absent in 100PLA (Figure 6B). Similar behaviour and the presence of the double melting peak were observed by the authors in biocomposites based on polylactic acid and olive wood waste, indicating a different behaviour during the phase transition between the polymer and the organic filler [35].

Leonard and Berrio, on the other hand, highlighting the same characteristic in the PLA-based filaments and cocoa bean shells they have developed and which have not undergone any chemical treatment, attribute the existence of two peaks during melting to the different degradation temperatures of hemicellulose and cellulose present in the filler [18]. This last interpretation seems to be in agreement with the DSC curve of CBSW obtained in our study and shown in Figure 4B.

### 3.3. Characterization of 3D Samples

**Figure 7:**
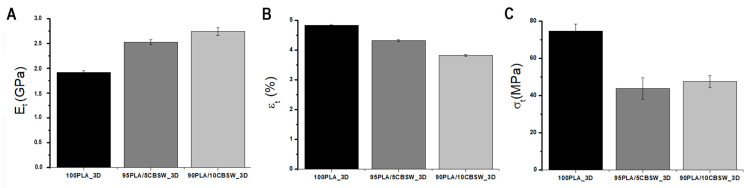
Mechanical properties from tensile tests of 3D samples: (**A**) Young’s modulus E_t_ (GPa), (**B**) elongation at break ε_t_ (%), (**C**) maximum strength (MPa).

Three-dimensional specimens for tensile testing were produced by FFF printing in accordance with ISO 527-2(2012) [26]. The overall average results are shown in Figure 7. The results of the pure PLA samples are consistent with those reported in the literature [1].

The Young’s modulus (Et) increased in the CBSW-based samples. Indeed, an inevitable consequence of the addition of filler to the polymer matrix is an increase in the viscosity of the polymer melt, which usually depends on the volume fraction of filler and the size and shape of the particles (Figure 7A) [36]. Overall, the presence of filler appears to have hindered the mobility of the polymer chain of the PLA matrix, thereby increasing the stiffness of the 3D sample composite. Furthermore, this increase appears to be linear, as observed by the Et value increasing with filler wt.%. It has been reported in the scientific literature that the addition of the filler actually modifies the polymer phase and the polymer interacts with the filler surface to form an adsorbed polymer interphase [36]. The thickness of this interphase (as well as the value of the Young’s modulus) depends on the bond between the polymer and the filler, which in turn depends on the surface area (i.e., the shape and size of the filler particles). Based on these considerations, the different behaviours of the composites could probably be due to a stronger interaction between the polymer and the filler in the 90PLA/10CBSW_3D sample, caused by the larger specific active surface area of the CBSW particles, as well as the higher volume fraction of the filler [36].

The elongation at break εt (%) always decreases with the addition of filler to pure PLA (Figure 7B), in agreement with the literature [17,30], leading to an increase in the fragility of the composites compared to the polymer. Similarly, the value of the maximum strength σt (MPa) decreases in both the 95PLA/5CBSW_3D and 90PLA/10CBSW_3D samples (Figure 7C) with a non-linear trend. This is probably due to a different orientation of the filler particles dispersed in the polymer matrix. Overall, the tensile test results show a lower ductility and a change in the mechanical properties of CBSW based composites; the non-uniform distribution of CBSW particles in the composite structure is the main cause.

**Figure 8:**
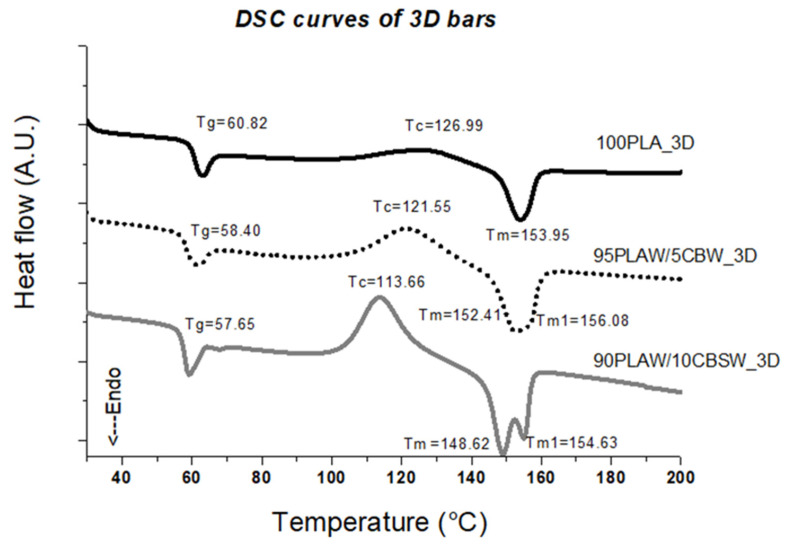
DSC curves of 3D samples.

The DSC analysis performed on the FFF printed 3D samples is shown in Figure 8. A decrease in glass transition temperature (T_g_) is observed for all 3D samples compared to the pure PLA 3D sample. The crystallization (T_c_) and melting temperatures (T_m_ and T_m1_) of the CBSW-based samples also decrease but remain higher than those measured for the corresponding composite filaments, indicating a greater interaction between the polymer chains and the filler and a higher degree of crystallinity after the printing process. This also explains the increase in Young’s modulus of the CBSW-based 3D samples described above. There is also always a double melting peak in 3D samples due to the presence of the filler in the polymer matrix, i.e., different phases.

**Figure 9:**
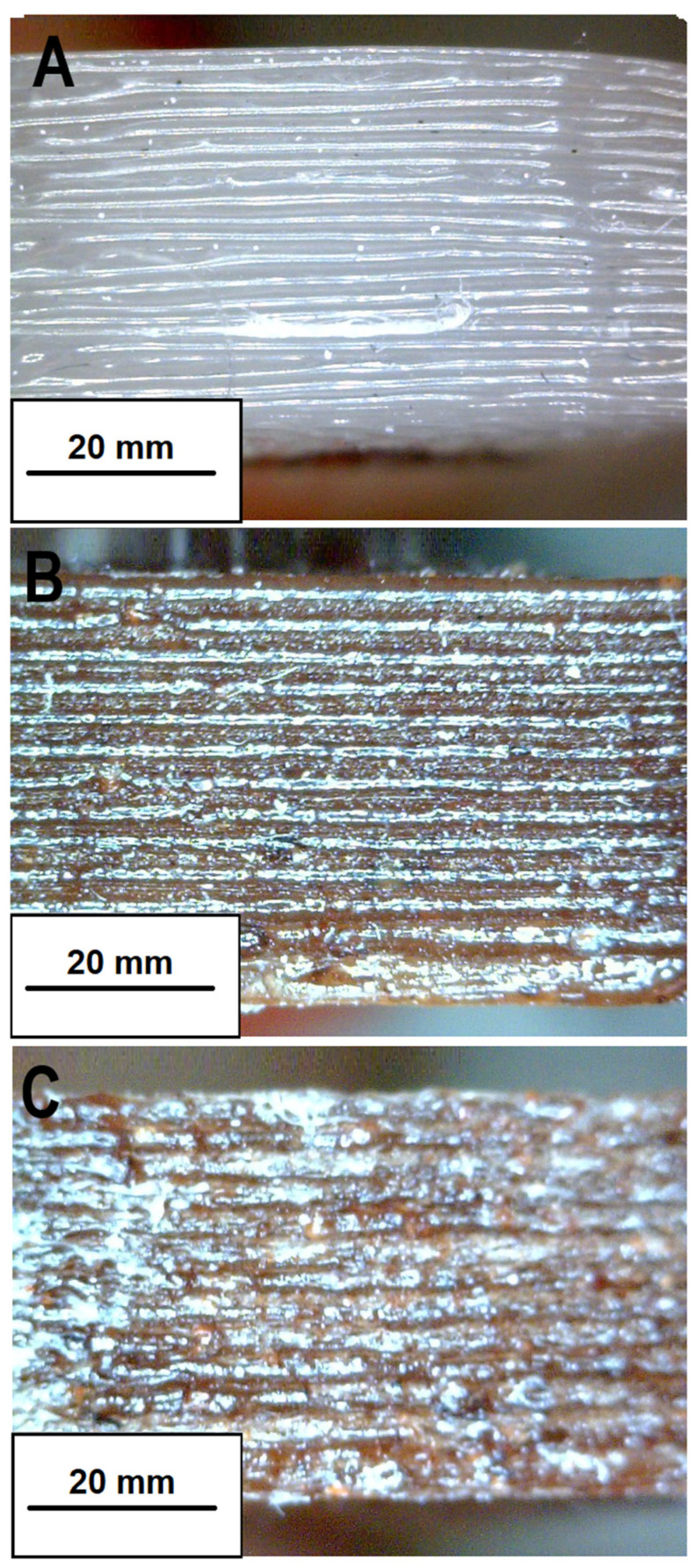
Magnified images (50ϗ) of the 3D bars: (**A**) printing surface of the 100PLA_3D specimen; (**B**) printing surface of the 95PLA/5CBSW_3D specimen; (**C**) printing surface of the 90PLA/10CBSW_3D specimen.

To better understand the printability of the developed biofilaments, a microscopic analysis was performed along the side surface of the 3D samples. The images obtained by light microscopy are shown in Figure 9 and overall show good adhesion between the 3D layers in both the 100PLA_3D sample (Figure 9A) and in the 95PLA/5CBSW_3D sample (Figure 9B), in contrast to the 90PLA/10CBSW_3D sample (Figure 9C). The addition of CBSW filler to the polymer matrix, which causes the formation of particle aggregates in the filament, causes adhesion problems between the layers in the 90PLA/10CBSW_3D; as in the other 3D samples, the layers are perfectly aligned with each other and do not show swelling, deformation, or detachment; however, the presence of some air voids is observed, and this porosity probably inhibits the mechanical performance of the composite material.

### 3.4. From Agro-Industrial Waste to New Objects

**Figure 10:**
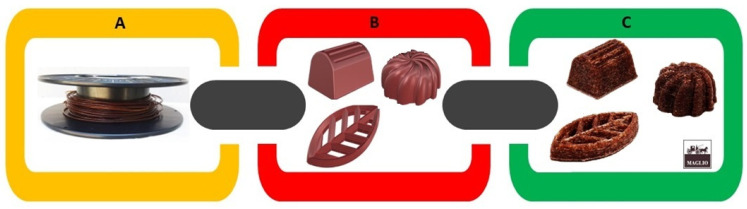
Manufactured filament (**A**), CAD model created with Rhinoceros version 7 software (Robert McNeel & Associates, Seattle, DC, USA) (**B**) and representative printed samples (**C**).

In this work, the waste materials, i.e., cocoa bean shell waste CBSW, were supplied as a residue from the chocolate manufacturing process in the form of a coarse-grained, non-homogeneous powder. These are residues from chocolate processing that the company must necessarily discard, with a significant time and economic effort. In this section, the authors want to illustrate an example of one of the many sustainable applications that can be imagined, i.e., reusing recycled materials to create a new object that can be redeployed by the same company that produced the waste. Based on the tested performance of the developed cocoa and poly(lactic acid) biofilaments, the 90PLA/10CBSW filament was selected for 3D printing the chocolate-shaped objects (prototypes) using Fused Filament Fabrication 3D printing technology (Figure 10). Specifically, the authors chose to produce 3D printed objects in the shape of traditional chocolates or other shapes, so that they could be used by the supplier company for display in the shop window or inside the shop as design and furnishing objects, having greater durability and resistance to environmental conditions than real chocolate. The final object after printing preserves the same environmental resistance characteristics as the 3D bars tested in the previous paragraph. Most interestingly is that already the 5 wt.% of cocoa powder (CSBW) product allows the organoleptic characteristics of pure chocolate to be preserved in the 3D object, giving customers the feeling that it is a real product exhibited in a store window.

## 4. Conclusions

In this study, novel green composite filaments for FFF were developed and characterized, utilizing cocoa bean shell waste and polylactic acid. Structural–morphological, thermal, and mechanical characterization of both raw materials and products (extruded filaments and 3D printed specimens) was comprehensively conducted. Overall, the findings revealed significant filler aggregates at 10 wt.% CBSW concentration in the PLA matrix. However, at both CBSW concentrations (5 wt.% and 10 wt.%), no notable morphological or thermal alterations were observed, and satisfactory printability was achieved. Moreover, tensile tests on the 3D printed objects demonstrated enhanced rigidity in these specimens and better load resistance at the highest CBSW concentrations. Finally, the biofilament exhibiting superior properties (containing the 10 wt.% of CBSW) was utilized to fabricate some simple 3D printed chocolate models, showcasing the efficacy of the proposed approach in successfully recycling agro-industrial waste within the same production company, leading to economic and ecological benefits. Future research endeavors aim to identify biomarkers confirming the longevity of cocoa organoleptic properties post-printing process and investigate the durability of biocomposites under controlled environmental conditions.

## Figures and Tables

**Table 1:** Extrusion process parameters for filaments.

Extrusion Process Parameters	100PLA	95PLA/5CBSW	90PLA/10CBSW
Screw speed (rpm)	3.5	4	4
Fan cooling speed (%)	0	30	30
Feed zone temperature (°C)	170	165	165
Compression zone temperature (°C)	185	180	180
Metering zone temperature (°C)	190	185	185
Die temperature (°C)	200	165	165

**Table 2:** Raw Materials, biofilament and 3D printed sample labels and weight composition (wt.%).

Label	Type	Composition (wt.%)
PLA	Raw material	100 PLA Ingeo 4043D pellet
CBSW	Raw material	100 Cocoa bean shell waste powder
100PLA	Filament	100 PLA Ingeo 4043D
95PLA/5CBSW	Filament	95 PLA Ingeo 4043D and 5 Cocoa bean shell waste
90PLA/10CBSW	Filament	90 PLA Ingeo 4043D and 10 Cocoa bean shell waste
100PLA_3D	3D sample	100 PLA Ingeo 4043D
95PLA/5CBSW_3D	3D sample	95 PLA Ingeo 4043D and 5 Cocoa bean shell waste
90PLA/10CBSW_3D	3D sample	90 PLA Ingeo 4043D and 10 Cocoa bean shell waste

## Data Availability

Data are contained within the article.
